# Hypoxic Modulation of HLA-G Expression through the Metabolic Sensor HIF-1 in Human Cancer Cells

**DOI:** 10.1155/2017/4587520

**Published:** 2017-07-11

**Authors:** Marica Garziera, Lucia Scarabel, Giuseppe Toffoli

**Affiliations:** Experimental and Clinical Pharmacology Unit, CRO Aviano National Cancer Institute, IRCCS, Via F. Gallini 2, 33081 Aviano, Italy

## Abstract

The human leukocyte antigen-G (HLA-G) is considered an immune checkpoint molecule involved in tumor immune evasion. Hypoxia and the metabolic sensor hypoxia-inducible factor 1 (HIF-1) are hallmarks of metastasization, angiogenesis, and intense tumor metabolic activity. The purpose of this review was to examine original in vitro studies carried out in human cancer cell lines, which reported data about HLA-G expression and HIF-1 mediated-HLA-G expression in response to hypoxia. The impact of *HLA-G* genomic variability on the hypoxia responsive elements (HREs) specific for HIF-1 binding was also discussed. Under hypoxia, HLA-G-negative cell lines might transcribe HLA-G without translation of the protein while in contrast, HLA-G-positive cell lines, showed a reduced HLA-G transcriptional activity and protein level. HIF-1 modulation of HLA-G expression induced by hypoxia was demonstrated in different cell lines. *HLA-G* SNPs rs1632947 and rs41551813 located in distinct HREs demonstrated a prominent role of HIF-1 binding by DNA looping. Our research revealed a fine regulation of HLA-G in hypoxic conditions through HIF-1, depending on the cellular type and *HLA-G* genomic variability. Specifically, SNPs found in HREs should be considered in future investigations as markers with potential clinical value especially in metastatic malignancies.

## 1. Introduction

Cancer immunotherapy was identified as Clinical Cancer Advance of 2016 at the latest American Society of Clinical Oncology (ASCO) annual meeting [1]. Its emerging role further emphasizes the importance of studying the impact of immune system regulation in cancer, in particular the influence of the nonclassical human leukocyte antigen-G (HLA-G) in the mechanisms involved in tumorigenesis. HLA-G is a nonclassical major histocompatibility complex (MHC) class I molecule, and it is thought to act as an immune checkpoint molecule [2]. Alternative splicing of the primary transcript may generate seven different isoforms, four membrane bound (HLA-G1 to HLA-G4) and three soluble (HLA-G5 to HLA-G7), all capable of exerting a negative regulation on immune cells such as natural killer (NK), cytotoxic T lymphocytes (CTLs), and antigen-presenting cells (APCs), by binding to specific receptors [3, 4]. Moreover, tumor-associated macrophages (TAMs), involved in tumor progression, angiogenesis, and suppression of antitumor immunity, express surface HLA-G and secrete or shed HLA-G molecules [5]. Two important mechanisms in the tumor escape from the host immune recognition and destruction are the expression of HLA-G and/or the complete loss or down regulation of classical HLA class I molecules [6, 7]. These phenomena explain why expression of HLA-G on cancer cells is associated with a higher tumor grade and a poor prognosis [8–10]. The escape from host immune system surveillance, the cancer immunoediting process, is considered one of the emerging hallmarks of cancer with relevant effects on patient's prognosis [11]. Additionally, some 3′ untranslated region (3′UTR) single nucleotide polymorphisms (SNPs) of the *HLA-G* gene were found to be independently associated with cancer susceptibility [12–14] and cancer prognosis [15, 16]. Several genetic variations involved in *HLA-G* regulation have been described also in the 5′ upstream regulatory (or promoter) region (5′URR or 5′UTR), while in contrast to the classical *HLA* class I loci, a lower variability in the coding regions is observed [17]. HLA-G is highly expressed in physiological conditions in immune privileged sites such as in trophoblast during placentation, at fetal-maternal interface, while in normal adult tissues has a restricted distribution [18]. Its expression can be induced in pathological conditions such as in autoimmune disorders and cancer [19, 20]. Many processes that involve cellular invasion, blastocyst implantation, placental development, and also rapidly growing tumors occur under reduced oxygen environments [21]. The dynamic and multifactorial process of hypoxia also activates signaling pathways leading to angiogenesis, enhanced motility/invasion, changes in metabolism, and the ability to survive to oxidative stress [22]. Hypoxia is known to increase the metastatic and angiogenic potential of tumor cells and has been associated with increased metastasis and poor prognosis in patients with different types of cancer [22–25]. Tumor cells are characterized by high proliferation rates finally resulting in structural and functional abnormal blood vessels unable to provide an adequate amount of oxygen to sustain the increased metabolic demands of cell growth, which ultimately results in hypoxia [22]. HLA-G expression can be modulated by epigenetic and tumor microenvironmental factors [26, 27] and induced in the presence of hypoxia and/or hypoxia-mimicking conditions [28–30]. The hypoxia-inducible factor 1 (HIF-1) is a heterodimeric transcription factor associated with hypoxia and hypoxia-mediated angiogenesis, metastasis, and resistance to chemo/radiotherapy, therefore with different tumor pathways [22, 23]. In particular, HIF-1 complex binds to hypoxia-responsive elements (HREs) sequences (5′-RCGTG-3′) in the promotorial region of hypoxia-response genes [31]. In the last years, HIF-1 was demonstrated to induce the transcription of *HLA-G* under hypoxia or hypoxia-mimicking conditions [30]. Some specific HREs were identified in *HLA-G* promoter and nonpromoter regions by in silico analyses, but only recently the robustness of these HIF-1 binding sites was characterized [32]. Binding of HIF-1 to an active HRE was assessed for a well-known cancer biomarker for cancer immunotherapy, the programmed death-ligand 1 (PD-L1), suggesting the close interaction between hypoxia and immune regulation [33]. Since HLA-G is considered an immune checkpoint molecule and a promising protein in cancer research as potential target for optimization of current cancer immunotherapy strategies, in the next sections, we reviewed the status of knowledge on HLA-G expression in hypoxic stress condition in human tumorigenic cell lines, focusing on the modulation exerted by the metabolic sensor HIF-1.

## 2. HLA-G Expression in Hypoxic Stress Conditions

Under physiological conditions, hypoxia is observed during placental development. In the early phases of pregnancy, extraembryonic tissue growth is of critical importance for a successful placentation [34]. The proliferative response to hypoxia allows trophoblast cells to thrive within the implantation site during the first 10 weeks of pregnancy [35]. A major challenge of trophoblasts is the constitutive high expression of HLA-G, and it is well accepted that maternal immune-tolerance is attributed to this molecule [36]. HLA-G expression is recognized to be an essential factor for successful embryo implantation that occurs under the hypoxic uterine atmosphere [36]. Moreover, HLA-G expression at the maternal-fetal interface is relevant because trophoblasts are devoid of the other classic MHC class I and II molecule expressions [37]. On the other hand, prolonged placental hypoxia is known to be a central pathogenetic factor in preeclampsia. In the preeclamptic placentae, the invasion of extravillous cytotrophoblasts into the uterine wall is shallow, and the role played by HLA-G at the maternal-fetal interface seems to be crucial in this pathogenesis [37, 38].

Besides the role of HLA-G in trophoblasts and placenta development, most human solid tumors contain hypoxic areas, and HLA-G expression has been documented in several types of cancer [20, 22]. Hypoxia is a common feature of rapidly growing malignant cells and their metastases, favoring angiogenesis, resistance to anticancer drugs and intratumoral inflammation [39]. It reflects not only the aberrant growth of proliferating cancer cells but also the abnormal vasculature network resulting in transient blood flow. In this review, we focused on human cancer cell lines. Among tumors, malignant melanoma was the first type of cancer in which *HLA-G* transcripts were detected since 1998 [40]. Chang and Ferrone [41] analyzed *HLA-G* mRNA levels in melanoma cell lines using as positive internal control the JEG-3 choriocarcinoma cell line that constitutively expresses higher levels of HLA-G (HLA-G^+^), both at the transcriptional and protein level. *HLA-G* transcripts were detected in 11 out of 16 cell lines at different levels (higher in FON-1 and OCM-1A), and five were negative (HLA-G^−^), such as 1074mel cell line. Anyway, paradoxical absence of the protein in each HLA-G^+^ cell line was observed. Using an in silico analysis, the authors for the first time identified a consensus HRE, at position −242 base pairs (bp) from the start ATG codon, assuming that *HLA-G* gene should be considered also as a stress-responsive gene [41]. Thus, influence of hypoxia on modulation of *HLA-G*, which is a physiologically relevant tumor-related stress gene, was investigated. *HLA-G* mRNA expression was induced in negative 1074mel after time-dose dependent assays in hypoxic conditions, using the hypoxia mimetic desferrioxamine (DFX). HLA-G expression could be detected as early as 3 hours post DFX-treatment, reaching the maximum level after 24 hours, even if this level was approximately 16-fold lower than the level of constitutive *HLA-G* mRNA in JEG-3. Mouillot et al. [30] confirmed these preliminary results in 1074mel cell line in the same experimental conditions. Furthermore, the authors verified the influence of hypoxia on HLA-G expression in different HLA-G^−^ tumorigenic cell lines such as melanoma (M8), choriocarcinoma (JAR), Burkitt's lymphoma (Raji), and glioma (U87MG, LN229, and LN428). *HLA-G* gene activation in M8 cell line started after 12 hours with higher transcript levels reached after 24 hours of incubation; anyways, similar to 1074mel cell line, they were lower than those detected in JEG-3 cells at the basal level. Patterns of alternatively spliced *HLA-G* transcripts in M8 were very similar to JEG-3 with the exception of a slight HLA-G3 isoform expression. As previously reported [41], HLA-G protein was not detectable with different methodological approaches in both M8 and 1074mel cells. Intriguingly, opposite effects in HLA-G expression in the presence of hypoxia-mimicking treatment were found in HLA-G^+^ melanoma FON and JEG-3 cells: transcript levels were decreased by an average of 67.8% and 38.5% in FON and JEG-3 cells, respectively. A kinetic of DFX treatment in JEG-3 demonstrated that the reduction of *HLA-G* transcription started after 6 hours of hypoxia and was still observed at 72 hours posttreatment in FON cells. Patterns of alternatively spliced *HLA-G* transcripts were not different. Moreover, after prolonged hypoxia conditions, HLA-G expression on cell surface in FON was reduced, even if all cells remained positive. In contrast, expression of membrane-bound HLA-G (mbHLA-G) was maintained in JEG-3 cells. Finally, besides the first identified [41], a novel HRE in the 5′UTR of *HLA-G* promoter was found by in silico analysis at position −966 before the ATG codon, although an in vitro validation was not performed [30]. The suggestion that *HLA-G* should be considered as a stress-responsive gene and was also tested exposing 13 EBV-transformed B cell lines to nutrient deprivation deficiency, to hypoxia, or to both stress conditions, to induce *HLA-G* [42]. A common feature of all these experimental settings was the variability in the percentage of HLA-G-positive cells evaluated by flow-cytometry (FC), because not all cells transcribed and expressed the protein to the same degree.

In particular, a moderate increase in mbHLA-G was observed in oxygen deprivation conditions simulated by flushing cell cultures with nitrogen gas (N_2_) and leaving cells with the stopper of the flask closed.

Hypoxic microenvironment plays also a key role in the progression of glioblastoma, a very aggressive tumor, in which HLA-G over-expression was well documented [43]. Recently, the impact of hypoxic stress in HLA-G expression in U251MG glioblastoma cell line was explored [32]. In this study, under hypoxia, the U251MG cells (HLA-G^−^) induced significant *HLA-G* transcriptional activity compared to untreated cells, but with negative protein detection. Transcription was increased of about 103-mean-fold when cells were treated with the demethylated agent 5-aza-dC (5-aza-2′deoxicytidine or decitabine), and this effect was further boosted when 5-aza-dC and DFX were combined. Luciferase reporter assay (LRA) confirmed that hypoxia stress induced by DFX produces and enhances transcriptional activity at the *HLA-G* promoter region in treated cells. Summary of modulation of HLA-G expression in tumorigenic cell lines and details on treatments in hypoxic stress conditions are presented in Table 1.

## 3. The Metabolic Sensor HIF-1

HIF-1 is a basic helix-loop-helix (bHLH) protein consisting of two subunits: a cytoplasmic oxygen-regulated HIF-1*α* isoform codified by hypoxia inducible factor 1 alpha subunit (*HIF1A*) gene, which is upregulated in dependence of oxygen concentration, and a nuclear constitutive HIF-*β* subunit [23]. In normoxia, HIF-1*α* is prolyl hydroxylated, interacts with the tumor suppressor Von Hippel-Lindau (VHL) protein, and becomes polyubiquitinated and finally degraded. Under hypoxia conditions, the prolyl hydroxylase (PHD) becomes nonfunctional and the stabilized HIF-1*α* could translocate into the nucleus and dimerizes with the HIF-1*β* subunit. The heterodimeric *αβ* transcription factor through the recognition and binding to HREs will activate the expression of hypoxia-response genes, also of the immune pathways [44]. Overexpression of HIF-1*α* has been observed in many cancer types and associated with a poor prognosis [45, 46]. Inhibition of HIF-1 activity by either anthracycline chemotherapy or acriflavine is known to prevent tumor vascularization in in vivo studies, suggesting that HIF-1 role is critical in tumor angiogenesis [47, 48]. Moreover, HIF-1 also favors the vascular endothelial growth factor A (VEGF-A) production by stabilizing its mRNA through the *VEGF* 3′UTR. HIF-1*α* upregulates the levels of cathepsin D (CTSD), urokinase-type plasminogen-activator receptor (uPAR), and matrix metalloproteinase-2 (MMP2) enzymes, implicated in the basement membrane disruption leading intravasation and dissemination of circulating tumor cells (CTCs) in the organism [45]. Moreover, HIF-1 showed a critical role in survival, inflammation, metabolism [47–50], and immunometabolism [51]. Immunometabolism embraces the idea that changes in metabolism truly regulate the phenotype and function of immune cells by controlling transcriptional and posttranscriptional events [52, 53]. Hypoxia, nutrient deprivation and immune cell activation may affect immunometabolism because immune responses are highly energy dependent [53]. Hypoxia and its metabolic sensor HIF-1*α* participate into the modulation of the immune system both in adaptive and innate responses [54–59].

## 4. Evidences of HLA-G Balanced Expression through HIF-1 in the Presence of Hypoxic Stress

The first demonstration that in hypoxic conditions the modulation of HLA-G expression is dependent on HIF-1 stabilization was achieved by Mouillot et al. [30]. To verify if the transcription factor HIF-1 is involved in the modulation of HLA-G expression, authors exposed HLA-G^−^ M8 cell line and HLA-G^+^ FON and JEG-3 cell lines, to DFX and to nitric oxide donor sodium nitroprusside (SNPr), an inhibitor of HIF-1*α* activation. Cytoplasmic stabilization of HIF-1*α* was detected at different incubation times in M8, JEG-3 (3 hours) and FON cells (6 hours) after DFX treatment. HIF-1 inhibition in M8, FON, and JEG-3 cells was observed in the presence of both DFX and SNPr after 6 hours. When DFX plus SNPr treatment was prolonged (24 hours), *HLA-G* transcription was still repressed in M8 cells but was increased in FON and JEG-3 cells. Therefore, upon DFX treatment, HIF-1 inhibition led to the return of the former basal level of *HLA-G* gene transcription, suggesting that HIF-1 has a role in the modulation of HLA-G under hypoxic conditions.

In addition to hypoxia, HIF-1*α* can also be activated in response to protoinflammatory mediators such IL-1*β* and plays a key role in glioma progression [60]. The authors previously reported that IL-1*β* induces HIF-1*α* in glioma cells through an IL-1*β*-HIF-1*α* feedback loop [61]. Increased levels of HLA-G expression, promoter activity, and surface protein expression were detected in glioma cells cultured with the addition of IL-1*β*. Glioblastoma cells exposed to IL-1*β* and transfected with a construct carrying HIF-1*α* siRNA led to the protein expression abrogation. These experiments demonstrated that IL-1*β* induces HLA-G in glioma cells in a HIF-1*α*-dependent manner [60].

**Figure 1:**
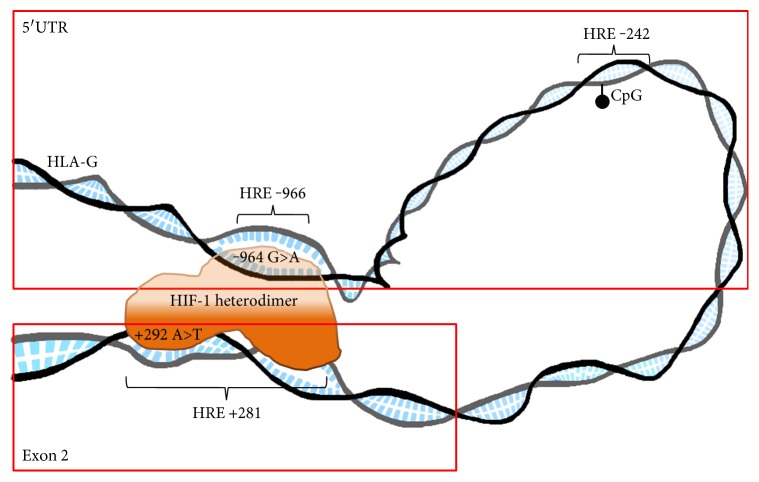
Schematic representation of the hypothesized interaction between hypoxia activated HIF-1 and *HLA-G* in −966 HRE and +281 HRE sequences specific for HIF-1 binding. Orange color highlights that +281 HRE is the primary site for HIF-1 binding while −966 HRE site participates to the creation of the *HLA-G*/HIF transcriptional complex through a DNA looping. *HLA-G* polymorphisms −964 G>A (rs1632947) and +292 A>T (rs41551813) located in the promoter and in the exon 2 regions, respectively, were evidenced for their possible influence on HIF-1 binding. Methylation at CpG site in −242 HRE present in the 5′UTR *HLA-G* promoter region was represented, as reported in different cell lines in the literature (HIF-1: hypoxia-inducible factor 1; HLA-G: human leukocyte antigen-G; HRE: hypoxia-responsive element; rs: reference sequence number; 5′UTR: 5′ untranslated region).

The impact of variability of *HLA-G* genomic sequence in influencing HIF binding in *HLA-G* target sites and thus in modulation of HLA-G expression under hypoxic conditions was explored for the first time by Yaghi et al. [32]. The authors found by in silico analysis, a new HIF-1 binding site located in the *HLA-G* coding sequence at exon 2, 281 base pairs after start nucleotide [32]. This novel +281 HRE comprehends two hypoxia binding sites (HBS), one at +281 bp (sense, 5′-ACGTG-3′) and one at +291 bp (antisense, 5′-CACGC-3′) positions [32]. The role of putative HIF target sites on *HLA-G* transcription in hypoxia was first analyzed by LRA for the two already reported −966 and −242 HREs [30, 41]. The first HRE in the promoter region of *HLA-G* contains a natural and common polymorphism, −964 G>A (rs1632947) that has been associated with asthma [62]. In the presence of the −964*G* allele in the first HRE region (−966(*G*)), luciferase activity was slightly affected by DFX and not significantly suggesting that HREs in the 1.4 kb HLA-G promoter region are not much relevant. Furthermore, electrophoretic mobility shift assay (EMSA) was also performed to determine if HIF-1 could bind to these HREs. HREs are subjected to oxidative damage leading the formation of an abasic site in one strand, and high sensitivity to oxidative damage of the terminal guanines in the HIF-1 target sequences has been reported [63]. EMSA, performed on −242 or −966(G) oligonucleotides with abasic sites, revealed that these *HLA-G* HREs were unable to bind HIF-1. In particular, competition experiments showed that *HLA-G* −242 alone was not functional, while *HLA-G* −966(G) alone had a slight, but not strong enough, affinity to transactivate *HLA-G* promoter in LRA. Subsequently, the impact of the novel putative HRE (named +281 HRE) was explored suggesting a major role of this *HLA-G* region in hypoxia conditions. Furthermore, significant enhanced activity in DFX treated cells with a 5-mean-fold enhancement in the presence of −966*A* and wt (wild type) exon 2 was measured, and a 10-mean-fold enhancement in the presence of wt −966*G* and wt exon 2. When scrambled sequences in exon 2 at +281 and +291 were created, and in the presence of −964*G* allele in the −966 HRE, luciferase activity was impaired, reinforcing the hypothesis that mutations in the *HLA-G* exon 2 HRE sequences may strongly influence the HIF-1 binding. Of note, EMSA assays demonstrated that +281 HBS and +291 HBS in the presence of DFX generated a specific complex containing HIF-1*α*. Chromatin immunoprecipitation assays (ChiPs) were carried out and demonstrated that stabilized HIF-1 was bound to exon 2 in *HLA-G* locus when cells were treated with 5-aza-dC and DFX. These culture conditions allowed both high levels of *HLA-G* gene induction and HIF-1*α* stabilization. Overall, these data demonstrated that the novel HRE in *HLA-G* exon 2 is the major target for HIF-1 binding induced by hypoxia (Figure 1). Anyway, the −966 HRE site participates to the creation of the *HLA-G*/HIF1 transcription complex through a DNA looping (Figure 1). Summary of modulation of HLA-G expression in tumorigenic cell lines exposed to HIF-1 inhibitors and details on treatments in hypoxic stress conditions are presented in Table 2.

**Figure 2:**
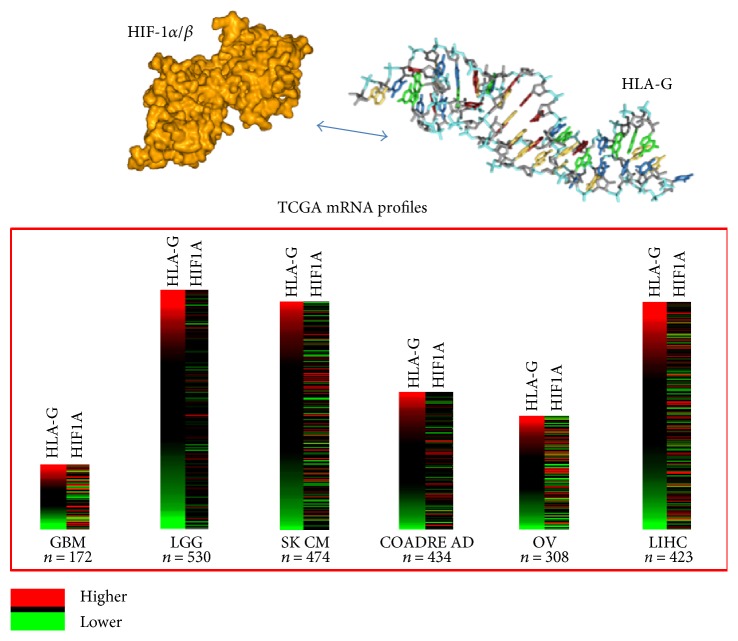
The HIF-1 active form/*HLA-G* interplay and heatmap representation of the TCGA mRNA profiles of *HLA-G* and *HIF1A* genes in 6 different TCGA cohorts. Data were extracted from public UCSC Xena browser (https://xenabrowser.net/): higher (red) and lower (green) expression levels suggest that *HLA-G* has a different and broad range of expression levels with respect to *HIF1A* (HIF-1: hypoxia-inducible factor 1; HLA-G: human leukocyte antigen-G; GBM: glioblastoma; LGG: lower grade glioma; SKCM: melanoma; COADREAD: colon and rectal cancer; OV: ovarian cancer; LIHC: liver cancer).

Furthermore, to better explore the interplay between *HLA-G* and *HIF1A* in human tumors, we performed an in silico analysis by using The Cancer Genome Atlas (TCGA) gene expression data. The exon expression profiles were detected using the Illumina HiSeq 2000 RNA Sequencing platform (polyA+IlluminaHiSeq) by the University of North Carolina TCGA genome characterization center and published on the public UCSC Xena browser (https://xenabrowser.net/). Data were extracted (Table 3) from Xena Browser considering gene expression RNASeq values from 6 distinct TCGA cohorts: glioblastoma (GBM), lower grade glioma (LGG), melanoma (SKCM), colon and rectal cancer (COADREAD), ovarian cancer (OV), and liver cancer (LIHC). Other relevant hypoxic gene markers such as forkhead box P3 (*FOXP3*), vascular endothelial growth factor A (*VEGFA*), and interleukin-17 (*IL17A*) were also considered. Median expression levels of *HLA-G* were about half compared to *HIF1A*, and *VEGFA* and similar to that reported for *FOXP3*, a key nuclear transcription factor induced by HIF-1 important in adaptive immune responses for regulatory T cells (Tregs) recruited at inflammatory hypoxic sites [64]. IL-17A is a hallmark of helper CD4^+^ T-cell type 17 lymphocytes that infiltrate human CRCs: the density of these cells has been correlated with a poor prognosis in CRC [65, 66]. *IL17A* expression levels were reported only for COAREAD cohort and were lower with respect to all the other markers. Particularly, we observed that expression range was wide for *HLA-G* in all the cohorts studied, especially in SKCM (2.02–12.8), while for *HIF1A*, the distribution was near to the median value such as in SKCM (9.25–12.9) suggesting a great variability in *HLA-G* expression for each tumor type of TCGA cohort (Figure 2).

## 5. Crucial Role of *HLA-G* Genomic Variability in HREs for HIF-1 Binding

The importance of having integral HREs for high-affinity HIF-1 binding was here investigated for the corresponding HREs in the promoter and coding sequence of *HLA-G* gene. The presence of SNPs (i.e., frequency of at least 1% in the population) or mutations (i.e., frequency less than 1% in the population) in these specific *HLA-G* regions which could possibly affect HIF-1 binding was searched for in public databases (http://browser.1000genomes.org, http://hla.alleles.org, and http://cancer.sanger.ac.uk/cosmic). Genomic position numbers refer to NG_0290039.1, which considers the adenine of the first translated ATG as nucleotide +1 (at position 5867 in NG_029039.1) [62], the beginning of exon 1. In the first hypoxia responsive region located in the 5′UTR of *HLA-G*, the −966 HRE, only the already mentioned 5′UTR −964 G>A SNP, was found. Considering the overall population according to 1000 Genomes project, consisting of 1092 individuals from 14 different populations, the −964 G>A SNP is very frequent, and minor allele frequency (MAF) is related to the *G* allele (0.457). Furthermore, the presence of *G* allele in position −964 before ATG has been associated with a series of distinct promoter (PROMO) haplotypes (PROMO-G010101_a-d_, PROMO-G0103_a,e_) that characterize *HLA-G* 5′UTR region [17, 62]. As suggested by the authors who first identified −966 HRE [30], this particular association with only a few *HLA-G* alleles supports the relationship with the heterogeneous responses upon hypoxic treatment among the different cell lines. The PROMO-G010101a is one of the most common promoter haplotypes with a frequency > 30% [62]. *HLA-G* promoter is very polymorphic, and promoter haplotype demonstrated that most of SNPs here presented are in complete linkage disequilibrium; thus, they are transmitted together during meiosis. Other nucleotide variations (excluding −964 G>A SNP) were not found in both −966 and −242 HREs, in the *HLA-G* gene. Considering *HLA-G* coding sequence, exon 1 codifies for the signal peptide (24 AA), while exons 2, 3, and 4 codify for the extraglobular *α*1, *α*2, and *α*3 domains, respectively. The *α*1 domain, which is present in each of the seven different possible spliced HLA-G isoforms, is constituted by 91 AA. Regarding the HRE in the exon 2 of *HLA-G* coding region, no nucleotide changes have been described for the first +281 HBS region, while in the second +291 HBS region, three nucleotide variations were reported. These variants lead to two missense changes and one synonymous change, all in codon number 31 of the HLA-G protein. The first nucleotide change is related to the exon 2 c.+292 A>T (rs41551813) SNP leading to a missense AA change from threonine to serine (T31S) in the *α*1 protein domain. This polymorphism is quite common since MAF related to mutated *T* allele is 5.4%. For the +292*T* mutated allele, four PROMO haplotypes (PROMO-G0103_d,a,e,e_) have been assigned with this change [17]. Two of them (PROMO-G0103_a,e_) are in common with −964*G* mutated allele even at a low frequency, 1.7% and 1.1%, respectively [62], and correspond to *HLA-G* alleles G^∗^01 : 03 : 01 : 01 and G^∗^01 : 03 : 01 : 02. The second alteration described, exon 2 c.+293 C>T, is a mutation (MAF (T) = 0.1%) which codes for another missense AA substitution from threonine to methionine (T31M). This mutation is represented in two *HLA-G* alleles, the G^∗^01 : 10 and G^∗^01 : 11. The last nucleotide variant reported, exon 2 c.+294 G>A, codes for the same type of AA, threonine (T31T); thus, it is a synonymous change. This is a somatic mutation detected in one patient affected by gastric cancer described in COSMIC (Catalogue of Somatic Mutations in Cancer) database, a very uncommon alteration. Summary of nucleotide changes reported in common databases for specific hypoxia responsive and binding regions for HIF-1 in the *HLA-G* noncoding and coding sequence is listed in Table 4.

## 6. Discussion

The adaptation of tumor cells to stress hypoxic environment leads to a more aggressive phenotype followed by regulation of various genes playing crucial roles in cellular proliferation, differentiation, tumor glycolysis, angiogenesis, and metastasis and invasion [45, 67, 68]. Indeed, the immune checkpoint molecule HLA-G contributes to the mechanisms used by tumor cells to differentiate towards cells with reduced immunogenicity. It is becoming apparent that the mechanisms controlling *HLA-G* gene activation have evolved to limit its expression, to control very specific functions in immune tolerance [26]. With this review, we pointed out a strict regulation of HLA-G expression in hypoxia conditions through HIF-1, depending on the cellular type. We observed opposite effects on *HLA-G* transcriptional activity when different tumor cellular types were exposed to hypoxia stress in comparison with normoxia conditions. HIF-1 in response to hypoxia acts as a negative or positive regulator of HLA-G depending on the type of cell line (HLA-G^−^ or HLA-G^+^), highlighting the delicate balance mediated by HIF-1 during adaptation of tumor cells to hypoxic environment. In HLA-G^+^ tumor cells, the amount of *HLA-G* transcripts was reduced (FON, JEG-3), while in HLA-G^−^ tumor cells, the *HLA-G* transcription was positively induced (1074mel, M8, U251MG). Normoxia conditions did not alter the pattern of basal *HLA-G* transcriptional and protein profile. HIF-1 modulation of *HLA-G* induced by hypoxia was demonstrated with the restoration of the basal expression level: HIF-1 inhibition during hypoxia led to a complete suppression of *HLA-G* transcription in HLA-G^−^ cell lines (M8, U251MG), and increased *HLA-G* transcript level in HLA-G^+^ cells (FON, JEG-3), after hypoxia stimulation. Similarly, the produced HIF-1 silencing diminished HLA-G protein expression in glioma cells (A17, U87MG) after IL-1*β* induction of HIF-1, even if in normoxia. *HLA-G* transcriptional activity in HLA-G^−^ tumor cells was not followed by a concomitant/simultaneous protein detection. Conversely, HLA-G^+^ tumor cells in the presence of hypoxia reduced *HLA-G* transcriptional activity and protein expression. Nevertheless, HLA-G was still present at the surface of these cells exposed to hypoxic treatment, especially in JEG-3, suggesting that the protective effect against immune system attack could be maintained and favor tumor growth. Otherwise, it is possible that HLA-G^+^ tumor cells may adapt to hypoxia directing the cell energy in productive gene expression at the expense of *HLA-G*, however maintaining the protection against host immune defenses carried by surface HLA-G expression [30]. Overall, these observations support the hypothesis that a HLA-G posttranscriptional regulation is present and may involve phenotypic in vivo factors present in the tumor microenvironment, especially when inflammatory processes are active [69]. Furthermore, the impact of polymorphisms present in *HLA-G* 5′ and 3′ untranslated sequences in influencing the response to endogenous cellular factors according to the cell type has been evidenced by the latest findings [70]. Epigenetic changes should also be not ignored as it is well documented that *HLA-G* gene activity is controlled by *cis*-acting epigenetic mechanisms, such as DNA methylation/demethylation and histone deacetylation/acetylation [26, 27]. Consistent with these observations is the activation of the *HLA-G* gene in tumor cell lines treated with the DNA-demethylating drug 5-aza-dC, a nonalkylating antitumoral agent [71, 72]. Methylation profile analyses in the 5′UTR *HLA-G* promoter (450 bp region before ATG) containing 19 CpG sites and the first identified −242 HRE for HIF-1 demonstrated that in HLA-G^−^ cells (M8, JAR), CpG methylation was dominant [27, 73]. Conversely, in HLA-G^+^ cell lines (FON, JEG-3), CpG sites were prevalently unmethylated [27, 73]. All the 19 CpG sites were methylated in the BG-1 ovarian adenocarcinoma cells (HLA-G^−^) and a region of 79 bp (−211 to −290) containing −242 HRE remained methylated after 5 days with 5-aza-dC 50 *μ*M treatment [74]. The authors hypothesized, due the observed negative regulation of HIF-1 on HLA-G^+^ cell lines FON and JEG-3, that methylation in HREs could prevent the binding of the potential repressor protein HIF-1 [74]. This phenomenon may help to maintain the transcription of *HLA-G* in ovarian tumors under hypoxic conditions, thus, allowing the tumor cells to evade cytotoxic T lymphocyte recognition and destruction [74]. Indeed, authors have hypothesized that in glioblastoma cells in hypoxic microenvironment culture conditions, CpG methylation in HIF target sites (5′RCGTG-3′) could moderate the observed *HLA-G* mRNA induction [32]. These findings suggest that the immunogenicity and tumorigenicity of various tumors may be potentially changed according antitumoral therapy with 5-aza-dC. The preliminary results on the already reported HREs (−966 bp, −242 bp) strongly urged that probably HIF binding sites were located outside the 1.4 kb promoter *HLA-G* sequence. Finally, a novel HRE (+281 HRE) in the coding exon 2 of *HLA-G* gene was individuated, and its strong impact on HIF-1 stabilization during hypoxia was established [32]. Although −966(*G*) *HLA-G* HRE alone was not able to bind HIF-1, it plays a role in *HLA-G* regulation in hypoxia-mimicking environment by DNA looping. Given the importance of *HLA-G* gene regions related to binding for HIF-1 in the hypoxia environmental contexture, we further explored the nucleotide variations in specific HREs for HIF-1 in *HLA-G* gene. These variants may explain in part the modulation in HLA-G expression found in different cell types; nonetheless, we should also not ignore the role of SNPs in the CpG islands that could be associated with variegated methylation profiles [75]. To this regard, we observed an inverse correlation between *HLA-G* and *HIF1A* mRNA expression and methylation profiles in the TCGA cohorts considered (data not shown). Differences in basal HLA-G expression described in human cell lines here studied were consistent with data reported in TCGA datasets Xena Browser for different types of tumor. The observed variability in HLA-G expression in each TCGA cohort and human cancer cell line (i.e., melanoma: M8/HLA-G^−^, 1074mel/HLA-G^−^, and FON/HLA-G^+^) examined could be related to the nucleotide genomic variations in cancer cell lines and patients with cancer, nucleotide variations in specific HREs for HIF-1 (during hypoxia conditions), and CpG methylation status in *HLA-G* 5′UTR.

We focused on the three reported HREs finding *HLA-G* nucleotide variants in these HIF-1 specific binding sites only in −966 and +281 HREs. Besides the most polymorphic −964 G>A SNP, another polymorphism was present at the exon 2 c.+292 A>T, codifying for a missense change (T31S). Considering the overall populations in 1000 Genomes project, the other two nucleotide variations susceptible to HIF-1 binding in +291 HBS are rare variants. Clustering of the 5′UTR −964 G>A SNP (rs1632947) and exon 2 c.+292 A>T SNP (rs41551813) could suggest a particular lineage in *HLA-G* PROMO haplotype [17, 62]. The presence of the minor allele *G* distinguishes six specific PROMO haplotypes in the 5′UTR *HLA-G* region [62]; among them, at least two (PROMO-G0103a, PROMO-G0103e) share the mutated *T* allele in the exon 2 (+292T) when serine AA is codified in codon 31 of translated HLA-G.

## 7. Conclusion

In conclusion, in this review, we highlighted the complex interplay between hypoxia and HIF-1 in control of HLA-G induction in human cancer cells. Giving the strong impact in HIF-1 binding and therefore on *HLA-G* transcriptional activity exerted by wt exon 2, in particular when associated with the 5′UTR −964*G* allele, we suggest that characterization of *HLA-G* regions in −966 and +281 HREs should be considered. The definition of these nucleotide regions could in part explain the different responses on *HLA-G* expression under hypoxia conditions observed among the tumor cell lines and TCGA cohorts, thus evidencing novel possible treatment strategies. It is well known that tumor progression and metastatic spread of cancer tissues have been related to changes in the levels of inflammatory mediators and increase oxidative stress [76]. Besides the acquisition of a more invasive and metastatic potential in response to hypoxia, tumor cells become more resistant to conventional treatments [22, 68]. Chemoresistance of cancer cells in locally advanced solid tumors develops because hypoxic regions are localized far from functional vasculatures; therefore, the diffusion and delivery of most anticancer drugs could be compromised as well as the cytotoxic effect [31, 77, 78]. Furthermore, alkylating agents and antimetabolites are also less effective under hypoxic conditions and cytotoxicity of some anticancer drugs is known to depend on molecular oxygen [22, 79]. Moreover, recent advances in molecular and cellular biology revealed an important role of HIF-1, in tumor radioresistance [80].

Overall, these observations suggest that a metastatic patient is particularly sensitive to environmental stress factors like hypoxia and oxidative stress, resulting into DNA damage upon exposure to a cytotoxic agent. Finally, we suggest that these *HLA-G* SNPs (rs1632947 and rs41551813) could be candidates as prognostic and/or predictive markers of response to chemotherapy treatment especially in metastatic malignancies and should be considered for the design of novel personalized therapeutic strategies.

## Figures and Tables

**Table 1:** Summary of modulation of HLA-G expression in human cancer cell lines exposed to hypoxic stress conditions.

Cell line	Basal HLA-G	Hypoxia induction by DFX (*μ*M)	5-aza-dC (*μ*M)	Incubation (h)	RT-PCR	WB (4H84)	FC (MEM-G/9)	LRA	Ref.
1074mel	HLA-G^−^	0, 150, 200, 250		3, 6, 12, 24	+	−^∗^			[41]
1074mel	HLA-G^−^	250		24	+	−^∗^	−		[30]
M8	HLA-G^−^	400		1, 3, 6, 12, 24	+	−^∗^	−		
JAR	HLA-G^−^	400		24	−				
Raji	HLA-G^−^	400		24	−				
U87MG	HLA-G^−^	400		24	−				
LN229	HLA-G^−^	400		24	−				
LN428	HLA-G^−^	400		24	−				
FON	HLA-G^−^	200		24, 48, 72	− −		−		
JEG-3	HLA-G^+^	200		1, 3, 6, 12, 24, 72	− −		±		
EBV-B	NA	N_2_		72					[42]
U251MG	HLA-G^+^	400		24	+	−	+	++	[32]
		400	100	72+ 24^∗∗^	++	++			

^∗^Antibody used was not specified; ^∗∗^U251MG cells were first treated for 72 hours with 5-aza-dC and secondarily with DFX; HLA-G, human leukocyte antigen-G; −, HLA-G-negative expression in response to hypoxia; +, HLA-G-positive expression in response to hypoxia; ++, HLA-G increased induction in response to hypoxia; − −, HLA-G decreased induction in response to hypoxia; ±, HLA-G unaltered induction in response to hypoxia; DFX, desferrioxamine; h, hours; *μ*M, micromolar; RT-PCR, reverse transcribed-polymerase chain reaction; WB, western blot; FC, flow cytometry; LRA, luciferase reporter assay; Ref., reference; NA, not available; N_2_, nitrogen gas; 5-aza-dC, 5-aza-2′deoxicytidine; 1074mel, melanoma; EBV-B, Epstein-Barr virus transformed B-cell; M8, melanoma; JAR, choriocarcinoma; Raji, Burkitt's lymphoma; U87MG, LN229, and LN428, glioblastoma; FON, melanoma; JEG-3, choriocarcinoma; EBV-B, Epstein Barr virus-transformed B; U251MG, glioblastoma.

**Table 2:** Summary of modulation of HLA-G expression in cell lines exposed to HIF-1 inhibitors in hypoxic stress conditions.

Cell line	HIF-1*α* stabilization	HIF-1 inhibitor	Treatment	Incubation (h)	RT-PCR	WB (4H84)	LRA	EMSA	ChiP	Ref.
M8	3 h DFX 400 *μ*M	SNPr 100 *μ*M	DFX + SNPr	24	− −					[30]
FON	6 h DFX 200 *μ*M	SNPr 100 *μ*M	DFX + SNPr	24	++					
JEG-3	3 h DFX 200 *μ*M	SNPr 100 *μ*M	DFX + SNPr	24	++					
A17	NA	HIF-1*α* siRNA 50 nmol/l	IL-1*β*^∗^ + HIF-1*α* siRNA	24		− −				[60]
U87MG	NA	HIF-1*α* siRNA 50 nmol/l	IL-1*β*^∗^ + HIF-1*α* siRNA	24		− −				
U251MG	3 h DFX 400 *μ*M	sh-HIF-1*α*	sh-HIF-1*α* + DFX 400 *μ*M	24	− −					[32]
			sh-HIF-1*α* + 5-aza-dC 100 *μ*M	72	− −					
			sh-HIF-1*α* + 5-aza-dC 100 *μ*M + DFX 400 *μ*M	72+ 24^∗∗^	− −					
			1.4 kb-5′URR (−966(A)) + DFX 400 *μ*M	24			±			
			1.4 kb-5′URR (−966(G)) + DFX 400 *μ*M	24			±			
			1.4 kb-5′URR (−966(G)) + DFX 400 *μ*M	3				±		
			1.4-WT exon 2 (−966(A)) + DFX 400 *μ*M	24			+			
			1.4-WT exon 2 (−966(G)) + DFX 400 *μ*M	24			++			
			1.4-WT exon 2(−966(G)) + DFX 400 *μ*M	3				+		
			1.4-WT exon 2 (−966(G)) + 5-aza-dC 100 *μ*M + DFX 400 *μ*M	72+ 3^∗∗^					+	
			1.4-MUT exon 2 (−966(G)) + DFX 400 *μ*M	24			±			

^∗^HLA-G transcription was induced by IL-1*β* treatment; ^∗∗^U251MG cells were first treated for 72 hours with 5-aza-dC and secondarily with DFX; HLA-G, human leukocyte antigen-G; −, HLA-G-negative expression in response to hypoxia; +, HLA-G-positive expression in response to hypoxia; ++, HLA-G increased induction in response to hypoxia; − −, HLA-G decreased induction in response to hypoxia; ±, HLA-G unaltered induction in response to hypoxia; HIF-1, hypoxia-inducible factor; NA, not available; *μ*M, micromolar; nmol, nanomole; RT-PCR, reverse transcribed-polymerase chain reaction; WB, western blot; LRA, luciferase reporter assay; EMSA, electrophoretic mobility assay; ChiP, chromatin immunoprecipitation assay; Ref., reference; DFX, desferrioxamine; SNPr, sodium nitroprusside; h, hours; IL-1*β*, interleukin-1*β*; siRNA, small interfering RNA; shRNA, short hairpin HIF-1*α*; 5-aza-dC, 5-aza-2′deoxicytidine; 5′URR, 5′ untranslated regulatory region; WT, wild type; MUT, mutated; M8, melanoma; FON, melanoma; JEG-3, choriocarcinoma; A172 and U87MG, glioblastoma; U251MG, glioblastoma.

**Table 3:** Summary of gene expression in different TCGA cohorts.

Gene	GBM		LGG		SKCM		COADREAD		OV		LIHC	
	*n*	Median (range)^∗^	*n*	Median (range)^∗^	*n*	Median (range)^∗^	*n*	Median (range)^∗^	*n*	Median (range)^∗^	*n*	Median (range)^∗^
*HLA-G*	172	3.97 (0–7.11)	530	4.62 (1.36–8.1)	474	6.87 (2.02–12.8)	434	6.77 (2.8–10.9)	308	4.15 (0–9.51)	423	6.15 (2.64–10.6)
*HIF1A*	172	12.4 (10.6–13.9)	530	12.1 (10.4–13.6)	474	11.1 (9.25–12.9)	434	11 (9.55–12.4)	308	11.7 (10.3–13)	423	10.5 (8.09–12.8)
*FOXP3*	172	3.27 (0.868–5.47)	530	3.24 (1.34–5.21)	474	5.69 (1.68–9.54)	434	5.28 (1.55–8.77)	308	5.7 (2.06–8.16)	423	5.11 (0–10.7)
*VEGFA*	172	12.7 (8.65–15.6)	530	9.29 (7.14–11.6)	474	9.88 (6.72–13.4)	434	11.6 (9.4–13.6)	308	12 (9.87–14)	423	11.7 (9.8–13.6)
*IL17A*	172	0 (0–0)	530	0 (0–0)	474	0 (0–0)	434	1.5 (0–5.91)	308	0 (0–0)	423	0 (0–0)

^∗^Values were obtained from public UCSC Xena browser (https://xenabrowser.net/) with Y coordinates: transform off and log scale log2(normalized_count+1); TCGA, The Cancer Genome Atlas; GBM, glioblastoma; LGG, low-grade glioma; SKCM, melanoma; COAREAD, colon and rectal cancer; OV, ovarian cancer; LIHC, liver cancer; *HLA-G*, human leukocyte antigen-G; *HIF-1A*, hypoxia inducible factor 1 alpha subunit; *FOXP3*, forkhead box P3; *VEGFA*, vascular endothelial growth factor A; *IL17A*, interleukin 17A.

**Table 4:** Summary of nucleotide variants found in HREs and in HBS for HIF-1 binding in *HLA-G* gene.

HRE in *HLA-G*	HBS in *HLA-G*	*HLA-G* region	Nucleotide position	Type of change	AA change	MMAF%	Ref. number	*HLA-G* PROMO haplotypes^∗^
−966 HRE	−966 HBS (5′-GCGTG-3′)	5′UTR	−964 G>A	5′UTR PROMO variant		45.7 (G)	rs1632947	PROMO-G010101a
								PROMO-G010101b
								PROMO-G010101c
								PROMO-G010101d
								PROMO-G0103a
								PROMO-G0103e
+281 HRE	+291 HBS (5′-CACGC-3′)	Exon 2	c.+292 A>T	Missense	(Thr31Ser)^∗∗^	5.4 (T)	rs41551813	PROMO-G0103d
								PROMO-G0103a
								PROMO-G0103e
								PROMO-G0103e
			c.+293 C>T	Missense	(Thr31Met)^∗∗^	0.1 (T)	rs72558173	NR
			c.+294 G>A	Synonymous	(Thr31Thr)^∗∗^	NR	COSM3861333	NR

^∗^According to the mutated minor alelle [62, 64]; ^∗∗^according to http://hla.alleles.org nomenclature; HLA-G, human leukocyte antigen-G; HRE, hypoxia-responsive element; HBS, hypoxia binding site; AA, amino acid; Ref, reference; c, nucleotide code position; rs, reference sequence number; MAF, minor allele frequency; UTR, untranslated region; Thr, threonine; Ser, serine; Met, methionine; NR, not reported.
